# A descriptive and bibliometric trend analysis of biological nursing research in the *Journal of Korean Biological Nursing Science* (2011–2025)

**DOI:** 10.7586/jkbns.26.013

**Published:** 2026-05-27

**Authors:** Hohyun Seong, Sungwon Lim, Jae Sim Jeong, Gyeong Ju An, Youn-Jung Kim

**Affiliations:** 1 College of Nursing, Chonnam National University, Gwangju, Korea; 2 Department of Nursing, Dongguk University WISE, Gyeongju, Korea; 3 Department of Clinical Nursing, Graduate School of Industry, University of Ulsan, Ulsan, Korea; 4 Department of Nursing, Cheongju University, Cheongju, Korea; 5 College of Nursing Science, Kyung Hee University, Seoul, Korea

**Keywords:** Bibliometrics, Nursing research, Biological science disciplines, Cluster analysis

## Abstract

**Purpose:**

This study provides an updated analysis of research trends in the *Journal of Korean Biological Nursing Science* (JKBNS) by examining changes in publication volume, methodological approaches, and thematic evolution among articles published between 2011 and 2025.

**Methods:**

Retrospective descriptive and bibliometric analyses were conducted on 537 articles published in JKBNS. Publication characteristics and statistical methods were summarized, and annual trends were examined. Keyword co-occurrence networks were generated using VOSviewer, and changes in research topics over time were explored using burst keyword analysis based on abstract-derived keywords.

**Results:**

Annual publication volume fluctuated but increased markedly in recent years, rising from 35 articles in 2024 to 58 articles in 2025. A language crossover occurred in 2024, when English-language articles (n = 19, 54.3%) exceeded Korean-language articles (n = 16, 45.7%); this predominance of English-language articles became more evident in 2025 (n = 36, 62.1%). Adult patients were the most common study population (n = 127, 23.7%). Analysis of variance was the most frequently used statistical method (n = 236, 43.9%), followed by linear regression (n = 84, 15.6%) and logistic regression (n = 59, 11.0%). Burst keyword analysis highlighted recent increases in aging- and health behavior–related terms. Co-occurrence mapping identified interconnected thematic areas spanning health behavior and risk, nursing knowledge and practice, and biologically oriented research.

**Conclusion:**

Between 2011 and 2025, JKBNS demonstrated growth in publication volume, a shift toward English-language articles, and evolving research topics, while maintaining thematic continuity with its biological orientation.

## INTRODUCTION

### 1. Background

Biological nursing research contributes to the advancement of nursing science by elucidating biological and physiological changes across the spectrum of health and disease and translating these insights into nursing interventions, education, and policy. Because such knowledge accumulates over time, the periodic synthesis of published studies is essential not only to describe what has been investigated but also to reveal gaps and inform future research priorities.

The *Journal of Korean Biological Nursing Science* (JKBNS), the official journal of the Korean Society of Biological Nursing Science, functions as a central venue for disseminating research grounded in biological nursing perspectives. The journal’s scope encompasses biological and physiological characteristics of individuals and environments, biological changes related to health and illness, methodological approaches relevant to biologically oriented nursing research, and interventions based on biological and physiological evidence [1]. As a journal’s readership and academic influence continue to expand, understanding how its published literature develops over time has become increasingly important for both contributors and users.

Contemporary healthcare environments are undergoing rapid demographic and epidemiological transitions, accompanied by advances in research methods and analytical technologies. For biological nursing, these changes are particularly meaningful because emerging health problems often require the integration of physiological indicators with behavioral and psychosocial constructs, as well as the evaluation of intervention mechanisms within complex clinical contexts [2,3]. Examining how such elements have been reflected in JKBNS publications can help clarify the evolving priorities and methodological profiles of the field.

A previous study provided a comprehensive trend analysis of JKBNS articles published from 1999, when the journal began publication, through to 2010 [4]. This analysis established an early baseline by summarizing the article characteristics, research designs, participant types, sampling strategies, measurement practices, and statistical approaches. It reported a substantial number of experimental and quasi-experimental studies, frequent reliance on convenience sampling, adult patients as the major participant group, and the common use of physiological indicators as outcome measures. The authors also noted a growth in publication volume during the journal’s early development and emphasized the need for continued strengthening of rigorous and practice-relevant biological nursing research. Since 2011, biological nursing research has expanded in volume and diversity within the context of population aging, changes in disease patterns, and evolving research infrastructure and dissemination systems [5-7]. These developments have prompted several questions regarding the journal’s recent trajectory: whether publication patterns, including language composition, have changed, whether study designs and analytic approaches have become more diverse, and which topical areas have recently gained momentum within the journal’s literature.

Bibliometric approaches offer a systematic framework for addressing these questions. In addition to describing the general characteristics of publications, bibliometric methods can illuminate the structure and evolution of a journal’s knowledge base by examining the relationships between keywords and temporal changes in their usage [8,9]. Keyword co-occurrence mapping helps visualize how major themes are interconnected through science mapping approaches [10], whereas burst detection identifies topics that show abrupt increases during specific periods, providing indicators of emerging or accelerating research interests [11].

Therefore, an updated analysis covering 2011–2025 is required to extend the earlier baseline and provide a contemporary profile of JKBNS publications. Such an investigation can support the assessment of how the journal has maintained thematic continuity with a biological orientation, while also identifying changes in publication patterns, methodological practices, and thematic emphases that may guide researchers, reviewers, and editors in decision-making.

### 2. Study aim

The aim of this study was to analyze all eligible articles published in JKBNS from 2011 to 2025 to (1) summarize publication and study characteristics and primary statistical methods, (2) describe annual trends in publication volume, language distribution, and participant groups, and (3) characterize topical dynamics using burst keyword analysis and keyword co-occurrence network mapping, thereby providing updated evidence on research trends in biological nursing within JKBNS in continuity with prior 1999–2010 analysis.

## METHODS

### 1. Study design

This study employed a retrospective descriptive design to map the publication patterns and research trends in JKBNS from 2011 to 2025. Using a bibliometric approach combined with structured content analysis, we summarized article characteristics and methodological features, and examined topical dynamics through burst detection and keyword co-occurrence networks.

### 2. Samples

The sample comprised all articles published in JKBNS between January 2011 and December 2025. To provide a comprehensive overview of the journal's publication trends, we included all published items without establishing exclusion criteria for specific article types. Consequently, the final analytic set of 537 articles consisted of original research articles, review articles, and editorials. Data collection was finalized on January 12, 2026, confirming that the complete 2025 volume (all issues) was fully published and included in the analysis.

### 3. Instruments

A structured data-extraction and coding form was developed to ensure consistent and reproducible classification of each article. To enhance continuity with prior JKBNS trend research and support longitudinal comparisons of research patterns, the coding scheme was informed by an earlier journal-level trend analysis covering 1999–2010 [4]. Building on this foundation, the form captured (a) publication characteristics (e.g., year, language, article type, and author role characteristics), (b) study features (e.g., participant group and sampling approach), (c) reporting-related items (e.g., whether reliability and validity information were reported), and (d) analytical information (e.g., primary analytical approach). Specifically, the coding of non-empirical articles was differentiated based on their methodological nature. Systematic and scoping reviews were categorized under "Literatures" for the research participant group, as their primary subjects are published studies. Conversely, other non-empirical items, such as editorials, were coded as "Others." For all such non-empirical publications, variables dependent on primary data collection (e.g., sampling method, reliability, and validity reporting) were systematically coded as "Not applicable." Additionally, fields were included to support text-based analyses by recording standardized keywords for burst detection and keyword co-occurrence mapping.

### 4. Data collection

Articles were retrieved from the official JKBNS journal website by compiling all issues published during the study period and downloading full-text files [12]. The retrieval process was completed on January 12, 2026, ensuring no additional articles for the 2025 volume would be published after our cut-off date. Two reviewers screened the records to confirm eligibility and verified the bibliographic information (e.g., year, volume/issue) against the journal archive to minimize missing or duplicate entries. Reference management was conducted using EndNote 20, and the full texts were stored in PDF format to facilitate systematic coding.

### 5. Data analysis

All eligible articles were coded in Microsoft Excel (Microsoft Inc., Redmond, WA, USA) using a predefined instrument. Two researchers independently coded the full set of articles, compared entries, and resolved discrepancies by re-checking the full texts and reaching a consensus to ensure coding reliability. Descriptive statistics were computed using SPSS Statistics version 25.0 (IBM Corp., Armonk, NY, USA) to summarize article characteristics.

Data visualization and processing were performed using Python within the Google Colaboratory environment, and keywords for burst analysis were extracted from article abstracts to ensure that annual document frequencies were calculated for each keyword. To prepare the abstract texts for this analysis, systematic preprocessing steps were applied. Initially, the texts were tokenized and converted to lowercase. Lemmatization was utilized to convert words to their base dictionary forms, thereby merging singular and plural forms and standardizing variant expressions. To maintain objectivity and ensure reproducibility, no external thesaurus-based synonym harmonization or additional manual keyword merging procedures were applied beyond this automated lemmatization. Standard English stopwords were subsequently removed. We opted to rely on standard stopword lists rather than manually filtering out general academic terms (e.g., experience, variance), as the sudden acceleration of such terms in abstracts can provide valuable signals regarding shifts in methodological reporting practices or broad conceptual emphases. Frequencies were standardized using z-scores to enable comparability across years. Specifically, the exact implementation of burst detection involved identifying "burst intervals," operationally defined as periods of one or more consecutive years where a keyword's annual z-score exceeded a threshold of 1.0 (i.e., one standard deviation above its 15-year mean frequency). The burst strength was then computed by summing the z-scores across all years within this continuous burst interval, providing a standardized metric for the magnitude of abrupt usage increases over time [11,13,14]. This operational definition was used to improve interpretability in abstract-based analyses, while remaining conceptually consistent with the established burst detection approaches used to identify emerging research trends.

A keyword co-occurrence analysis was performed using VOSviewer version 1.6.20 (Centre for Science and Technology Studies, Leiden University, Leiden, Netherlands). These science mapping approaches were executed in accordance with established bibliometric methodologies to ensure analytical rigor [9,10]. The full counting method was applied for this analysis. To focus on the most prominent research themes and prevent visual clutter in the network, the minimum occurrence threshold for keywords was set to five. During the network construction process, generic methodological terms (e.g., study, purpose, method) that lacked specific relevance to biological nursing research were manually excluded to preserve the thematic validity of the clusters. Network normalization was conducted using the association strength method. Co-occurrence networks were generated for the overall period (2011–2025) and for three consecutive 5-year intervals (2011–2015, 2016–2020, and 2021–2025). Node size represents keyword frequency, link thickness reflects co-occurrence strength, and the total link strength is used to summarize the overall connectedness of each keyword within the network [15].

### 6. Ethical considerations

This study analyzed previously published journal articles and did not involve new data collection or direct interactions with human participants; therefore, it was exempt from an Institutional Review Board review. All sources were cited appropriately, and eligibility screening and data extraction were conducted using predefined procedures. To minimize bias, two independent reviewers assessed article eligibility and performed coding; disagreements were resolved by discussion and consensus.

## RESULTS

### 1. Descriptive characteristics of published articles in JKBNS

Table 1 summarizes the general characteristics of the 537 articles published in JKBNS between 2011 and 2025. Most articles were published in Korean (n = 447, 83.2%), whereas 90 (16.8%) were published in English. In terms of study origin and funding, independent research was the most frequent (n = 225, 41.9%), followed by grant-funded studies (n = 187, 34.8%) and theses/dissertations (n = 125, 23.3%). Regarding authorship, the first author was most commonly a professor in a nursing school (n = 294, 54.7%), followed by nurses (n = 121, 22.5%) and graduate students (n = 105, 19.6%). Overwhelmingly, the corresponding author was a professor at a nursing school (n = 498, 92.7%), whereas authorship by nurses (n = 4, 0.8%) and graduate students (n = 16, 3.0%) was relatively uncommon.

Across the research participant groups, studies most frequently focused on adult patients (n = 127, 23.7%). A substantial proportion targeted the general population, including adults (n = 92, 17.1%) and university students (n = 77, 14.3%). Studies involving healthcare providers (e.g., nurses) accounted for 70 articles (13.0%), whereas literature-based papers comprised 50 articles (9.3%). Research using animals represented 22 articles (4.1%), and relatively few studies were classified as cell-based (n = 3, 0.6%) or microorganism-based (n = 2, 0.4%). With respect to sampling, convenience sampling was the most frequently reported approach (n = 334, 62.2%), followed by random sampling (n = 70, 13.0%); sampling was coded as not applicable in 67 articles (12.5%). The reporting of measurement quality indicators varied; reliability was reported in 279 articles (52.0%) and not reported in 107 articles (19.9%), whereas validity evidence was reported in 90 articles (16.8%) and not reported in 296 articles (55.1%).

### 2. Analytical approaches used in published articles

The distribution of the primary analytical approaches assigned to each article is shown in Supplementary Table 1. Analysis of variance (ANOVA) (including repeated measures ANOVA and analysis of covariance) was the most frequently used method, accounting for 236 articles (43.9%), followed by linear regression, which was reported as the primary analytical method in 84 articles (15.6%). t-tests (independent and paired) were utilized in 82 articles (15.3%), and logistic regression was employed in 59 articles (11.0%).

Other methods appeared less frequently, including literature review (n = 26, 4.8%) and correlation analysis (n = 8, 1.5%). Non-parametric tests (Mann–Whitney U or Wilcoxon) were used in 10 articles (1.9%), and the chi-square/Fisher’s exact test was used in three (0.6%). Advanced modeling approaches, including structural equation modeling/path analysis (n = 5, 0.9%) and factor analysis (n = 4, 0.7%), were relatively rare.

### 3. Trends in the number of published papers

#### 1) Annual publication volume and language distribution

**Figure 1.**
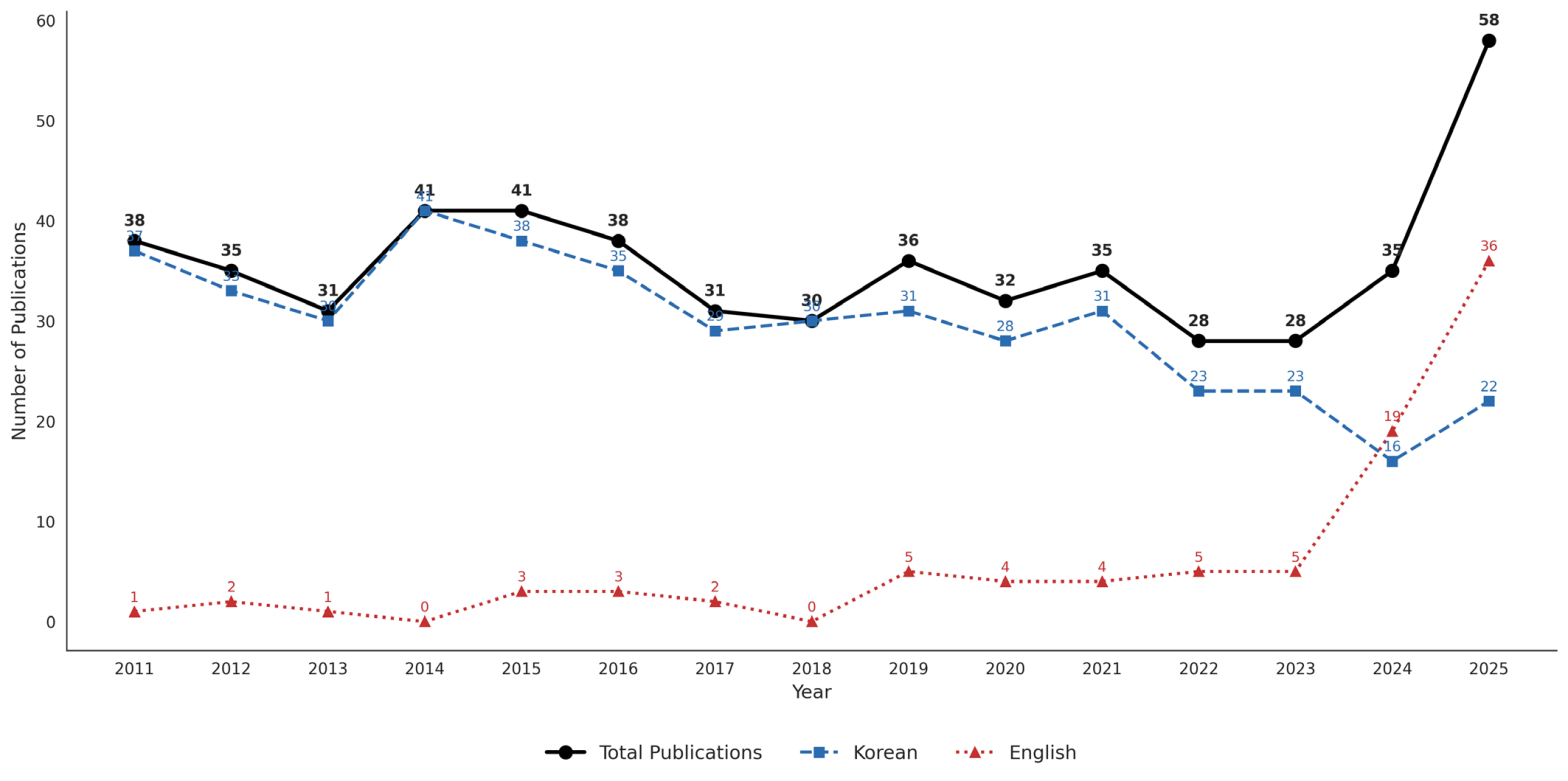
Annual publication volume and language distribution in *Journal of Korean Biological Nursing Science* (2011–2025). The solid line indicates the total number of articles published each year; dashed and dotted lines denote Korean- and English-language articles, respectively. Values above the markers indicate annual counts.

The annual publication volume and language distribution of JKBNS are shown in Figure 1. The overall publication output fluctuated across the study period but rose clearly in the most recent years. From 2011 to 2015, the number of published papers declined from 38 (2011) to 31 (2013) and then increased to 41 in 2014 and 2015. During 2016–2020, the annual total remained within a relatively narrow range (30–38 papers: 38 in 2016, 31 in 2017, 30 in 2018, 36 in 2019, and 32 in 2020). In 2021–2025, there were 35 (2021) and 28 in 2022 and 2023, respectively, followed by an increase to 35 in 2024 and a pronounced rise to 58 in 2025.

Language-specific patterns have also changed substantially over the final two years. Korean-language articles comprised the majority of publications from 2011 to 2023 (37 of 38 in 2011 and 23 of 28 in both 2022 and 2023), whereas English-language articles remained limited (0–5 articles per year through 2023). In 2024, the two language trends crossed, with 19 English-language articles (54.3%), exceeding the 16 Korean-language articles (45.7%). This shift became more pronounced in 2025, when English-language publications increased to 36 (62.1%) and Korean-language articles accounted for 22 (37.9%).

#### 2) Annual trends in research participants by group

**Figure 2.**
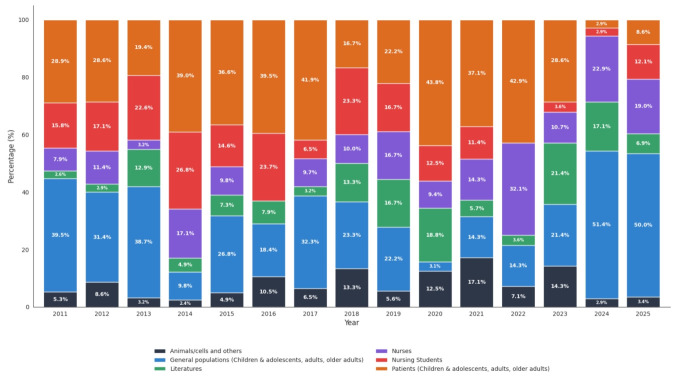
Annual distribution of study participant groups in *Journal of Korean Biological Nursing Science* (2011–2025). Bars show the proportion of articles by participant category (100% stacked). Categories include patients, general populations, nursing students, nurses, literature-based studies, and animals/cells/other; labels within bars indicate annual percentages.

Figure 2 summarizes the annual proportional distribution of research participants across six categories (patients, general population, nursing students, nurses, literature, animals/cells, and others). Overall, patients and the general population accounted for the largest proportions in most years, although their relative proportions varied across the study period. From 2011 to 2013, studies on the general population represented the largest segment (39.5% in 2011, 31.4% in 2012, and 38.7% in 2013), whereas patients comprised 28.9%, 28.6%, and 19.4%, respectively. From 2014 to 2017, the proportion of patient-focused studies increased (from 36.6% to 41.9%) and remained high from 2020 to 2023, reaching 43.8% in 2020 and 42.9% in 2022.

In contrast, the share of studies involving the general population has increased over the last two years, reaching 51.4% in 2024 and 50.0% in 2025. The proportions of the other participant groups also showed year-to-year variation. Studies involving nursing students ranged from 3.6% (2023) to 26.8% (2014), whereas those focusing on nurses ranged from 3.2% (2013) to 32.1% (2022). The proportion of literature-based studies increased from 2.6% (2011) to 21.4% (2023). Studies categorized as animals/cells and others ranged from 2.4% (2014) to 17.1% (2021), with higher proportions observed in 2018 (13.3%), 2020 (12.5%), and 2023 (14.3%). This category accounted for 2.9% of publications in 2024 and 3.4% in 2025, indicating a decrease in its relative proportion in recent years.

### 4. Burst keyword analysis

**Figure 3.**
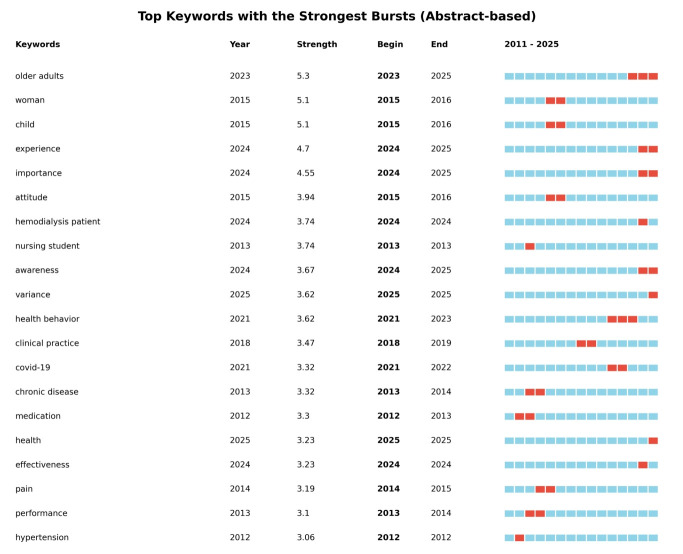
Top 20 abstract-derived keywords with the strongest bursts identified from *Journal of Korean Biological Nursing Science* publications (2011–2025). Red segments indicate the time periods during which each keyword showed a burst, and light blue segments represent non-burst periods.

Burst detection identified 20 keywords with the strongest temporal surges in abstract-based usage between 2011 and 2025 (Figure 3). The keyword with the highest burst strength was “older adults” (strength = 5.30), with a burst interval spanning 2023–2025. High-intensity bursts were also observed in the mid-2010s: “woman” (strength = 5.10) and “child” (strength = 5.10) both concentrated in 2015–2016, alongside “attitude” (strength = 3.94) during 2015–2016. Several burst keywords appeared in the earlier years of the study period, including “hypertension” (strength = 3.06, 2012), “medication” (strength = 3.30, 2012–2013), “nursing student” (strength = 3.74, 2013), “chronic disease” (strength = 3.32, 2013–2014), “performance” (strength = 3.10, 2013–2014), and “pain” (strength = 3.19, 2014–2015). In later years, bursts were identified for “clinical practice” (strength = 3.47, 2018–2019), “COVID-19” (strength = 3.32, 2021–2022), and “health behavior” (strength = 3.62, 2021–2023).

In the most recent period, multiple keywords showed concentrated bursts in 2024–2025, including “experience” (strength = 4.70, 2024–2025), “importance” (strength = 4.55, 2024–2025), and “awareness” (strength = 3.67, 2024–2025). Single-year bursts were also detected for “hemodialysis patient” (strength = 3.74, 2024), “effectiveness” (strength = 3.23, 2024), “variance” (strength = 3.62, 2025), and “health” (strength = 3.23, 2025).

### 5. Bibliometric analysis of keywords

**Figure 4.**
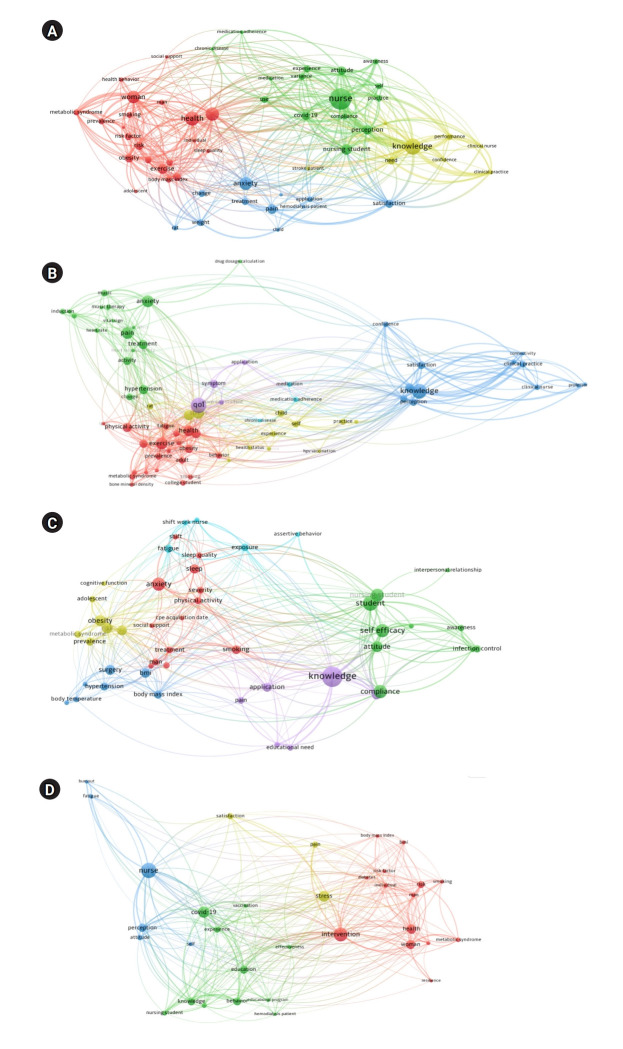
Keyword co-occurrence networks of *Journal of Korean Biological Nursing Science* publications illustrating the thematic structure and its evolution over time. Networks are shown for (A) the full period (2011–2025) and for three consecutive 5-year intervals: (B) 2011–2015, (C) 2016–2020, and (D) 2021–2025. Each node represents a keyword, with larger nodes indicating more frequently used keywords. Links between nodes indicate co-occurrence within the same articles, and thicker links reflect stronger co-occurrence. Colors denote clusters of keywords that tend to appear together, highlighting major thematic areas and changes in their relative prominence and interconnections across time windows.

Figure 4 visualizes the keyword co-occurrence networks for the entire period (2011–2025; Figure 4-A) and three consecutive 5-year intervals (Figure 4-B,C,D). Node size reflects keyword frequency, and line thickness reflects link strength.

#### 1) Keyword co-occurrence network for 2011–2025

Across the entire period, the most frequently used keywords were nurse, knowledge, health, and adult (Supplementary Table 2). Among the top keywords, knowledge showed the strongest overall connectedness (total link strength), followed by nurse, indicating broad co-occurrence relationships across topics. Several interconnected thematic areas were evident in the overall network map. One prominent area was centered on health and closely related terms reflecting health behaviors and risks, including keywords such as obesity, exercise, and body mass index. Another major area was organized around nurses and knowledge, accompanied by practice- and education-related terms, such as nursing student, perception, attitude, and satisfaction. The keyword “COVID-19” also appeared among the most frequent terms and was connected to other core topics in the network. In addition, a smaller sub-cluster in the lower-left region included biologically oriented terms, such as rat, weight, and change, indicating the presence of animal-model and physiological change–related keywords within the overall network.

#### 2) Keyword co-occurrence networks by 5-year intervals

Across the 5-year interval networks, a core set of frequently linked terms persisted, whereas the relative prominence of specific topics shifted over time. Between 2011 and 2015, the network concentrated on knowledge- and practice-related terms alongside health-focused areas, including health, exercise, and obesity. Between 2016–2020, education-related and behavioral terms (e.g., student, self-efficacy, and attitude) became more visible alongside health behaviors and symptom-related concepts. Between 2021–2025, coronavirus disease 2019 (COVID-19) emerged as a prominent node linked to nurses and education- and behavior-related keywords, with intervention- and risk-related terms present within the network structure.

## DISCUSSION

This study synthesized 15 years of research published in JKBNS from 2011 to 2025 by integrating descriptive characteristics, primary statistical methods, annual publication patterns, burst keyword detection, and keyword co-occurrence mapping. Across this period, JKBNS maintained thematic continuity with a foundational biological orientation, while also demonstrating discernible changes in publication volume, language composition, and the organization of research topics. Interpreting these findings alongside the journal’s earlier trend analysis covering 1999–2010 provides a useful longitudinal baseline for understanding how JKBNS has evolved in scope, methodological practice, and thematic emphasis across successive publication periods.

### Trends in the published articles and keywords

Several characteristics of the published articles appear to be stable when compared to the 1999–2010 period. An earlier analysis identified adult patients as the most common participant group and noted the frequent use of physiological indicators as outcome measures, with exercise interventions often represented in experimental studies [4]. From 2011 to 2025, adult patients remained the largest single-participant category, and a substantial proportion of studies targeted the general population of adults and university students. Taken together, this suggests continuity in a clinically relevant, health-centered orientation, along with greater visibility of population-based and health-promotion-oriented work within the biological nursing frame. In addition, the continued presence of literature-based studies and biologically oriented work, including animal/cell-related categories, indicates that JKBNS continues to host both evidence synthesis and mechanistic or experimental perspectives, which can be complementary when linked through clearly articulated nursing relevance. In terms of publication volume, JKBNS published 537 articles during 2011–2025, which is comparable to that reported for the Journal of the Korean Academy of Nursing Administration (506 articles, 2012–2021) [16] but lower than that reported for the Journal of the Korean Academy of Nursing (JKAN) (809 articles, 2010–2019) [17]. In Korean nursing specialty society journals, the numbers reported for shorter observation periods are generally smaller (e.g., 323 articles in the Journal of Korean Academic Society of Adult Nursing, 2010–2014; 153 articles in the Journal of Korean Gerontological Nursing, 2010–2015) [18,19], which likely reflects differences in journal aims and scope, publication frequency, and the specific years examined. Taken together, these cross-journal counts help situate the overall volume and continuity of JKBNS publications within Korean nursing scholarship; however, they should not be interpreted as direct evidence of relative growth, quality, or influence.

The methodological patterns also suggest an incremental evolution across periods. From 1999 to 2010, the commonly reported analytical approaches were descriptive statistics, t-tests, chi-square tests, and ANOVA. From 2011 to 2025, ANOVA remained prominent, but regression-based analyses, including linear and logistic regressions, were also frequently used as primary methods. This shift is notable in biological nursing research because multivariable modeling supports a more explicit estimation of associations and effects when behavioral, clinical, and physiological variables are examined together. Nevertheless, the relatively limited representation of advanced modeling approaches suggests that further methodological diversification is warranted in future work, particularly for questions involving mechanisms, longitudinal change, or clustered data structures.

Annual publication trends show year-to-year variability, with the highest output observed in 2025, followed by an increase from 2024 to 2025. A particularly notable shift is the language crossover in 2024, when English-language articles outnumbered Korean-language articles, with the predominance of English-language publications becoming more pronounced in 2025. While our bibliometric data clearly document the timing and magnitude of this language crossover, the underlying drivers were not directly measured in this study. We hypothesize that this recent shift might be associated with the journal's broader internationalization efforts, potentially including its Scopus indexing and evolving editorial strategies. Speculatively, institutional or journal-level incentives, along with authors' intentions to increase global discoverability, might have also contributed to this trend. However, these remain plausible hypotheses that require further empirical verification beyond the scope of this bibliometric review.

Burst keyword detection and keyword mapping together provide a higher-resolution view of topical change, capturing not only what appears frequently, but also what accelerates rapidly during specific intervals. In the burst analysis, “older adults” exhibited the strongest burst concentrated in 2023–2025, suggesting intensified attention to aging-related issues in recent years. Bursts related to COVID-19 and health behaviors in later years highlight how emerging societal and healthcare conditions can quickly be integrated into the journal’s research agenda. Additionally, multiple burst terms concentrated in 2024–2025, such as experience-, awareness-, and importance-related constructs, suggest that recent publications may be more actively engaging with subjective or psychosocial constructs, potentially in combination with biological or clinical variables, rather than focusing solely on disease labels or single-outcome indicators. When interpreted in light of the earlier period, during which physiological indicators and exercise interventions were prominent, these burst patterns suggest continuity in health-oriented biological nursing, while reflecting an expanding set of contexts and constructs through which biological nursing questions are articulated. As burst strength in this study operationalizes standardized year-by-year acceleration, burst findings should be interpreted as markers of rapidly increasing attention rather than direct indicators of overall topic dominance. Importantly, aging-related prominence is also visible in other Korean nursing journal trend analyses: for example, JKAN’s 2010–2019 keyword summaries included “Aged” and “Depression” among high-frequency terms in multiple years [17], indicating that population aging and related symptom/mental-health concerns have been persistent cross-journal themes even before the recent acceleration detected in JKBNS.

The co-occurrence maps further illustrate the journal’s thematic structure and are central to interpreting how JKBNS’s research areas relate to each other. In the overall map for 2011–2025, keywords were organized around two broad, interconnected axes: a health-focused area reflecting health behaviors and risk, and a nursing/knowledge-centered area associated with education and practice constructs. The prominence and interconnection of these axes are informative because they suggest that JKBNS has not developed separate streams of biological and nursing topics; rather, biologically oriented health themes appear to be embedded within a broader thematic structure that also emphasizes education, practice, and behavioral constructs. This integrated configuration is consistent with widely recognized directions in contemporary nursing science, which emphasize linking biological processes with behavioral, psychosocial, and contextual determinants of health within a unified research agenda [20]. It is also aligned with international strategic priorities that encourage cross-disciplinary integration across behavioral and biological sciences, including symptom science and self-management research that connects mechanisms, experiences, and care-related contexts [21]. Overall, the co-occurrence structure depicts an integrated knowledge base in which biologically oriented health questions are closely linked to education-, practice-, and behavior-related themes.

In the interval maps, the core terms persisted, whereas the thematic emphasis and connections shifted. Education- and behavior-related concepts became more visible in the middle interval, and COVID-19 emerged more distinctly in the most recent interval, indicating that newer contexts were integrated into established thematic areas rather than being displaced. This pattern is also consistent with global publication trends in nursing during the pandemic, where COVID-19 rapidly became a prominent topic across nursing journals and was often studied in relation to existing concerns such as practice, education, and workforce or behavioral responses [22]. Notably, the overall network also included a smaller biologically oriented sub-cluster containing terms such as rat, weight, and change, indicating that animal-model and physiological change–related research remained within the journal’s thematic landscape. This observation aligns with the journal’s biological nursing scope and complements earlier findings that highlight physiological indicators and biologically grounded interventions as prominent components of published research. Taken together, the interval-specific shifts and persistent biological subclusters suggest thematic expansion over time, retaining some biological orientation within a broadening scope of nursing research.

### Potential future trends in JKBNS

The comparison between the earlier baseline and the 2011–2025 period indicates that JKBNS is well-positioned to strengthen its international profile in biological nursing while sustaining a clear disciplinary focus. The recent prominence of age-related bursts, coupled with sustained health behaviors and risk-related clusters, supports the continued emphasis on research addressing older adults and chronic conditions, ideally integrating physiological pathways with nursing-relevant outcomes. Moreover, the coexistence of a robust nurse/knowledge axis and a health risk/behavior axis suggests an opportunity to strengthen research that explicitly connects knowledge and practice constructs to biological outcomes; for example, clarifying mechanisms of action in interventions and integrating behavioral, clinical, and physiological measures within coherent conceptual frameworks.

Looking forward, biological nursing research will be increasingly conducted within healthcare systems, shaped by rapid technological and epidemiological transitions. As digital health, wearable sensing, AI-assisted analytics, and technology-enabled interventions become more common, biological nursing is particularly well placed to evaluate how these innovations influence physiological regulation, symptom trajectories, and patient-centered outcomes across clinical and community settings [23,24]. In this context, JKBNS can further strengthen its role by encouraging translational lines of work that connect experimental insights, including animal model findings, to human intervention development and clinical application, while also supporting high-level evidence synthesis to consolidate rapidly expanding knowledge. Finally, the recent shift toward English-language publications suggests a strategic opportunity to expand the journal’s global contribution by prioritizing internationally harmonized terminology, transparent reporting, and reproducible analytic workflows–approaches that can enhance both scientific credibility and international reach while preserving the journal’s biological nursing focus.

This study has several limitations that should be acknowledged. First, the analysis was restricted to a single journal (JKBNS). While JKBNS is the premier representative journal for biological nursing in Korea, the findings may not fully capture the broader, global trends in the field or research published by Korean scholars in international journals. Second, while two researchers independently coded the articles and resolved all discrepancies through rigorous discussion and consensus, formal inter-rater reliability statistics (e.g., Cohen's kappa) were not calculated during the initial independent coding phase. This omission limits the quantitative verification of the initial coding consistency. Third, bibliometric analyses, particularly keyword co-occurrence networks and burst detection, are inherently sensitive to text preprocessing choices (e.g., lemmatization, stopword removal) and the specific parameters set within the software (e.g., minimum occurrence thresholds). Although we employed systematic and transparent procedures, different methodological parameters could potentially yield slight variations in the visual network structures.

## CONCLUSION

This study examined research published in JKBNS from 2011 to 2025 using descriptive profiling, statistical classification, temporal trend analysis, burst keyword detection, and keyword co-occurrence mapping. Overall, JKBNS demonstrated thematic continuity with a foundational biological orientation, with a persistent emphasis on health-related topics and adult populations, while showing clear changes in publication patterns and thematic structures over time. The journal’s publication profile demonstrated a recent increase in annual output and a notable language crossover in 2024, with English-language articles becoming predominant between 2024 and 2025. Topic analyses indicated that research attention has increasingly accelerated toward aging-related themes in recent years, while COVID-19 and health behaviors have emerged as time-sensitive focal areas. Keyword co-occurrence mapping further showed a connected knowledge structure organized around health risk/behavior and nursing knowledge/practice, with an additional biologically oriented sub-cluster reflecting animal models and physiological change–related research.

Together, these findings suggest that JKBNS is evolving toward greater international visibility while continuing to serve as a disciplinary platform for biologically grounded nursing scholarship. Future research published in JKBNS may benefit from strengthening translational connections between mechanistic and human studies, integrating behavioral, clinical, and physiological indicators within coherent conceptual frameworks, and addressing emerging priorities in aging societies and post-pandemic healthcare environments.

## Figures and Tables

**Table 1.** General Characteristics of Articles Published in JKBNS (2011–2025) (N = 537)

Variables		n (%)
Language		
Korean		447 (83.2)
English		90 (16.8)
Study origin and funding		
Independent research (non-funded)		225 (41.9)
Grant-funded		187 (34.8)
Thesis / Dissertation		125 (23.3)
First author		
Professor in nursing school		294 (54.7)
Nurse		121 (22.5)
Graduate student		105 (19.6)
Other		17 (3.2)
Corresponding author		
Professor in nursing school		498 (92.7)
Nurse		4 (0.8)
Graduate student		16 (3.0)
Other		19 (3.5)
Research participants		
Patient	Children and adolescents	4 (0.8)
	Adults	127 (23.7)
	Older adults	21 (3.9)
Family		1 (0.2)
General population	Children and adolescents	14 (2.6)
	University student	77 (14.3)
	Adults	92 (17.1)
	Older adults	42 (7.8)
Health care providers (nurses, etc.)		70 (13.0)
Literature		50 (9.3)
Animal		22 (4.1)
Cell		3 (0.6)
Microorganisms		2 (0.4)
Others		12 (2.2)
Sampling method		
Random sampling		70 (13.0)
Convenience sampling		334 (62.2)
Quota sampling		27 (5.0)
Purposive sampling		39 (7.3)
Not applicable		67 (12.5)
Reliability of instrument		
Yes		279 (52.0)
No		107 (19.9)
Not applicable		151 (28.1)
Validity of instrument		
Yes		90 (16.8)
No		296 (55.1)
Not applicable		151 (28.1)

JKBNS = *Journal of Korean Biological Nursing Science*.

## Supplementary Materials

Supplementary Table 1. Distribution of Primary Analytical Approaches Used in Published Articles

Supplementary Table 2. Most Frequently Used Keywords
