# Supplementary material for: A descriptive and bibliometric trend analysis of biological nursing research in the Journal of Korean Biological Nursing Science (2011–2025)
Source: J Korean Biol Nurs Sci. 2026 May 27;28(2):217–30. doi: 10.7586/jkbns.26.013 (PMC13463012; doi:10.7586/jkbns.26.013)
Supplement: Supplementary file 1 [file jkbns-26-013-Supplementary-Table-1.pdf]

Supplementary Table 1. Distribution of Primary Analytical Approaches Used in Published Articles (N = 537)

Analytical approach	n (%)
Parametric statistics	
t-test (independent, paired)	82 (15.3)
ANOVA (RM ANOVA, ANCOVA, etc.)	236 (43.9)
Linear regression (multiple, stepwise)	84 (15.6)
Pearson correlation	8 (1.5)
Non-parametric statistics	
Chi-square test (Fisher's exact test)	3 (0.6)
Non-parametric tests (Mann-Whitney, Wilcoxon)	10 (1.9)
Advanced/multivariate modeling	
Logistic regression	59 (11.0)
SEM and path analysis	5 (0.9)
Factor analysis (EFA, CFA)	4 (0.7)
Other analytical approaches	
Qualitative research (content analysis)	10 (1.9)
Literature review (systematic, scoping review, etc.)	26 (4.8)
Descriptive statistics	10 (1.9)

ANOVA = Analysis of variance; RM ANOVA = Repeated measures ANOVA; ANCOVA = Analysis of covariance; SEM = Structural equation modeling; EFA = Exploratory factor analysis; CFA = Confirmatory factor analysis.

https://doi.org/10.7586/jkbns.26.013
