# Supplementary material for: A descriptive and bibliometric trend analysis of biological nursing research in the Journal of Korean Biological Nursing Science (2011–2025)
Source: J Korean Biol Nurs Sci. 2026 May 27;28(2):217–30. doi: 10.7586/jkbns.26.013 (PMC13463012; doi:10.7586/jkbns.26.013)
Supplement: Supplementary file 2 [file jkbns-26-013-Supplementary-Table-2.pdf]

Supplementary Table 2. Most Frequently Used Keywords

Rank	Keyword	Occurrences	Total link strength
1	nurse	285	2,044
2	knowledge	202	2,366
3	health	174	1,236
4	adult	162	1,247
5	anxiety	152	704
6	woman	149	1,197
7	nursing student	118	804
8	pain	109	625
9	exercise	107	855
10	perception	104	1,139
11	obesity	94	908
12	COVID-19	93	768
13	attitude	92	852
14	BMI	86	700
15	risk	86	762
16	satisfaction	85	966
17	risk factor	78	512
18	need	76	951
19	weight	73	553
20	experience	71	643

COVID-19 = Coronavirus disease 2019; BMI = Body mass index.

https://doi.org/10.7586/jkbns.26.013
